# Chitosan from shrimp shell waste as a carrier for frankincense nanoparticles with enhanced antimicrobial activity

**DOI:** 10.1038/s41598-025-32342-x

**Published:** 2026-01-07

**Authors:** Habiba A. Ahmed, Zeinab A. Salama, Abeer E. Abd El-Wahab, Ahmed M. Aboul-Enein, Amr Nassrallah

**Affiliations:** 1 Plant Biochemistry Department, National Research Centre, Dokki, Giza, 12622 Egypt; 2 Medical Biotechnology Department, Genetic Engineering and Biotechnology Research Institute, City of Scientific Research and Technological Applications, New Borg El-Arab City, Alexandria 21934 Egypt; 3 Pharmaceutical and Fermentation Industries Development Center, City of Scientific Research and Technological Applications (SRTA-City), New Borg EL-Arab, Alexandria 21934 Egypt; 4 Biochemistry Department, Faculty of Agriculture, Cairo University, Giza, 12613 Egypt; 5 Basic Applied Science Institute, Egypt-Japan University of Science and Technology (E-JUST), P.O. Box 179, New Borg El-Arab City, Alexandria 21934 Egypt

**Keywords:** Chitosan purification, Frankincense extract, Nanoparticles, Antimicrobial activity, Biochemistry, Biological techniques, Biotechnology, Chemistry, Materials science, Microbiology, Nanoscience and technology

## Abstract

This study focuses on the sustainable extraction of chitosan from shrimp shell waste and its application in developing frankincense-loaded chitosan nanoparticles with enhanced antimicrobial efficacy. Chitosan was extracted through demineralization with 4% HCl, deproteinization using 8% NaOH at 70 °C, and deacetylation with 40% NaOH under shaking for 48 h, yielding 3.4 g (22.51%) from 15 g of shrimp shell powder. Frankincense ethanolic extract was incorporated into chitosan nanoparticles using sodium tripolyphosphate as a crosslinker, followed by ultrasonication and dropwise addition of chitosan to form a stable nanocomposite. Characterization confirmed a semi-crystalline structure (XRD), typical thermal degradation (TGA), and strong molecular interaction between chitosan and frankincense (FTIR). TEM and SEM showed well-dispersed, amorphous nanoparticles with smoother surfaces, while DLS revealed an average particle size of 149.4 nm, PDI 0.26, and zeta potential + 12.0 mV, indicating moderate stability. Antimicrobial activity was evaluated using two methods: the well diffusion and microplate reader assays against *Staphylococcus aureus*, *Streptococcus mutans*, *Escherichia coli*, *Salmonella typhi*, and *Candida albicans*. Results showed that nanoparticles significantly enhanced inhibition of *S. mutans*, *S. typhi*, and *C. albicans* compared to the extract. These findings highlight chitosan–frankincense nanoparticles as a promising natural antimicrobial system for pharmaceutical and food preservation applications.

## Introduction

Chitosan is a naturally derived polysaccharide widely recognized for its biocompatibility, biodegradability, non-toxicity, and inherent antimicrobial properties, making it valuable in biomedical, pharmaceutical, and environmental applications. It is produced through the partial deacetylation of chitin, a fibrous polysaccharide abundant in the exoskeletons of crustaceans, insects, and the cell walls of fungi^1^. Structurally, chitin is composed of 2-acetamido-2-deoxy-D-glucopyranose units linked by β-(1→4) glycosidic bonds, and deacetylation of these units yields chitosan with varying degrees of flexibility and reactivity.Industrial production of chitosan typically involves three major steps: demineralization, deproteinization, and deacetylation of crustacean shell waste such as shrimp, crab, and prawn shells^2,3^. The functional properties of chitosan particularly its solubility, charge density, and adsorption capability are strongly influenced by the degree of deacetylation, which determines the proportion of protonated amino groups along the polymer chain. These amino groups (pKa 6.2–7.0) are readily protonated in acidic conditions, allowing chitosan to dissolve in acids such as acetic, hydrochloric, nitric, or phosphoric acid, while remaining insoluble in neutral water^4^. Beyond biomedical applications, chitosan has gained significant attention in environmental remediation due to its strong affinity for organic and inorganic pollutants. Recent studies have demonstrated that chitosan-based materials, particularly magnetically functionalized chitosan nanoparticles, exhibit excellent adsorption efficiency for removing hazardous organic contaminants from wastewater. For example, Meena et al. (2017)^5^ reported the effective adsorption of cresol and its derivatives using magneto-chitosan nanoparticles, highlighting chitosan’s potential as a sustainable and efficient adsorbent in wastewater treatment. Growing interest in extracting chitosan from fishery waste aligns with global efforts to reduce environmental pollution and valorize marine by-products. The chitosan obtained from such sustainable sources presents considerable promise for advanced applications, including encapsulation technologies, drug delivery systems, and wastewater purification^6^. Boswellia sacra, a species from the Boswellia genus found mainly in northeastern Africa, the Arabian Peninsula, and the Indian subcontinent, is native to southern Oman, particularly the Dhofar region. This small tree produces frankincense, a fragrant oleo-gum resin obtained by making incisions in the trunk. Widely valued across cultures, frankincense is traditionally used in religious rituals and holds a significant place in folk medicine for treating various conditions such as skin disorders, digestive issues, liver problems, and arthritis^7^. Boswellia species, especially B. sacra, show various medicinal benefits, including analgesic, antioxidant, cardioprotective, and anti-inflammatory effects^8,9^. They also exhibit antimicrobial activity against bacteria and fungi, such as *S. aureus*, *E. coli*, and *Candida albicans*, and inhibit aflatoxin-producing fungi like *Aspergillus* species^10,11^. Researchers have increasingly focused on the use of phytoconstituents due to their therapeutic efficacy and minimal side effects^12^. Acetyl-11-keto-β-Boswellia acid (AKBA), a principal bioactive compound derived from *Boswellia serrata*, is classified as a Biopharmaceutical Classification System (BCS) Class IV drug due to its low aqueous solubility and limited membrane permeability^13^. These physicochemical limitations pose significant challenges in achieving effective therapeutic levels. To overcome these barriers, nanocarrier-based drug delivery systems have emerged as a promising strategy, offering improved solubility, enhanced bioavailability, and greater stability factors that collectively contribute to increased therapeutic efficacy^14^. Numerous studies have demonstrated the potential of nano-formulations as effective drug delivery systems for phytoconstituents^15–17^. In recent years, nanoparticles have garnered significant attention as a promising approach for enhancing oral drug delivery. Upon oral administration and contact with gastrointestinal (GI) fluids, nanoparticles typically ranging from 10 to 200 nm in diameter exhibit a high surface area-to-volume ratio, which facilitates improved dissolution, permeation, and stability of encapsulated drugs. This nanoscale size enables enhanced mucosal adhesion, transcellular transport, and protection of the active compound from enzymatic degradation, thereby improving the oral bioavailability of poorly water-soluble drugs^18–20^. Nanoparticle-based delivery systems have been extensively employed for the formulation of various phytoconstituents, including curcumin^21^, cinnamon oil^22^, morin^16^, and thymoquinone^23^. Multiple studies have demonstrated that nanoparticles can enhance the anticancer activity of both conventional chemotherapeutic agents^20^˒^24^ and phytoconstituents^25,26^. The physicochemical performance of nanoparticles is influenced by numerous factors, including the properties of the encapsulating polymer or lipid matrix, the nature of the bioactive compound, the particle size and surface charge, and the processing conditions during synthesis. These parameters collectively determine the stability, drug loading efficiency, release kinetics, and biological performance of the nanoparticle formulation^27–30^. This study applied a sustainable strategy to convert shrimp shell waste into chitosan and investigated its use as a carrier for *Frankincense* ethanolic extract in nanoparticle form. The aim was to enhance the antimicrobial efficiency of both natural materials while promoting the valorization of marine by-products. The prepared nanoparticles were characterized using FTIR to confirm chemical interactions, SEM and TEM to observe morphology and particle size, and dynamic light scattering (zeta sizer) to assess size distribution, surface charge, and stability.

## Materials and methods

All chemicals used in this study including hydrochloric acid (HCl), sodium hydroxide (NaOH), ethanol, sodium tripolyphosphate (TPP), and agar were of analytical-grade purity and suitable for laboratory research applications. These reagents were obtained from Sigma (USA) and Fluka (Switzerland). Frankincense was sourced from a local market and finely ground to ensure a uniform, homogeneous sample.

- **Preparation of chitosan** Three consecutive steps were used to extract and purify chitosan, including shrimp shells demineralization, Chitin processing (Deproteinization), and Chitosan processing (Deacetylation). In brief, collected shrimp shells (≈ 15 g) were dried in an oven at 40 °C for 48 h. The crisped shells were ground into fine powder, which was placed in opaque plastic bottles and stored at ambient temperature^31^.
- **Demineralization of shrimp shells** The produced fine powder of shrimp shell was demineralized with 4% HCl (v/v) under shaking at room temperature. After 24 h, the acid was removed by rinsing with water and dried in an oven at 50 °C.
- **Deproteinization** To obtain chitin, a demineralized step was carried out by deproteinizing with NaOH solution 8% (w/v) with stirring for 4 h at 70 °C. The remaining powder was washed with distilled water to remove NaOH and dried in an oven at 50 °C to obtain chitin.
- **Deacetylation** To obtain chitosan, the dried powder from the previous step was deacetylated. The chitin was rinsed into NaOH 40% solution (w/v) with shaking for 48 h at room temperature. Finally, the powder was rinsed with distilled water and dried at 50 °C.

### Chitosan yield determination

The yield of chitosan was calculated based on the weight of the chitosan obtained after the deacetylation process relative to the initial weight of shrimp shell powder used. After completion of the extraction and purification steps, the dried chitosan was weighed, and the yield was determined using the following equation:

$${\text{Yield (\% ) = Ws/Wc}} \times {\mathrm{100}}.$$

Where Ws is the weight of the chitosan obtained (g), and Wc is the initial weight of shrimp shell powder (g). There is a slash next to the percentage inside the parentheses in the equation that needs to be removed

### Preparation of chitosan-Frankincense NPs

**Fig. 1:**
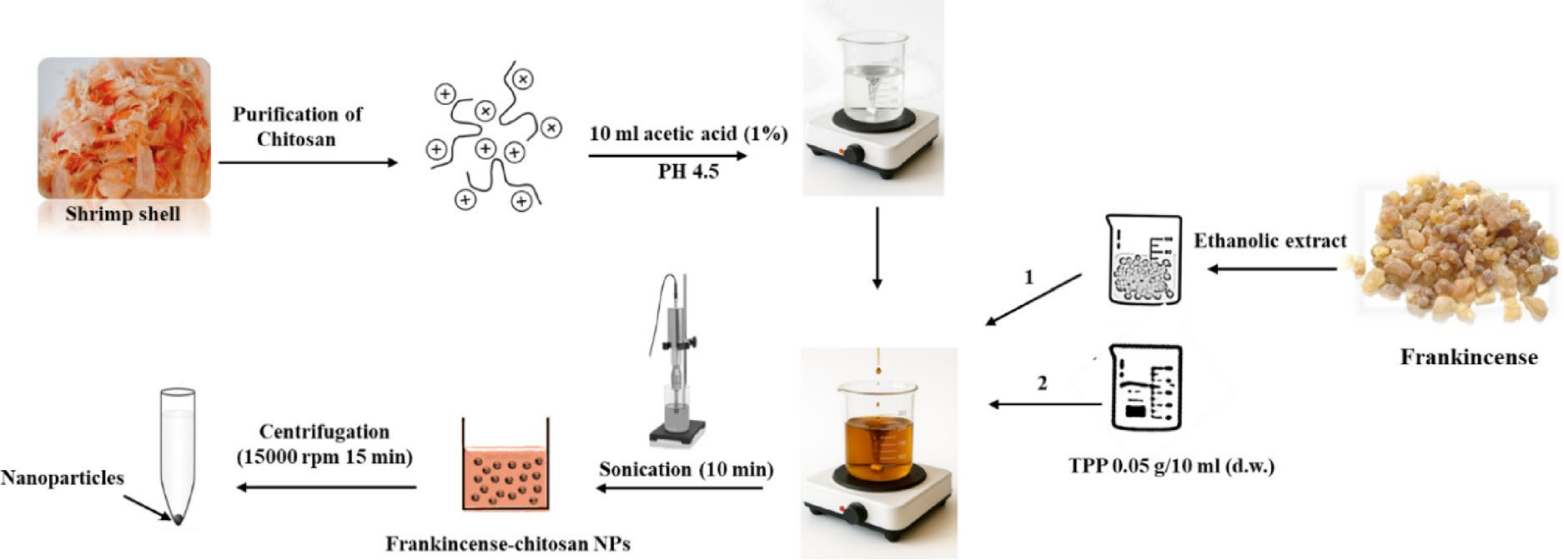
Schematic representation of the formation process of frankincense–chitosan nanoparticles.

Chitosan nanoparticles loaded with frankincense ethanolic extract (Fig. 1)^32^ are prepared by first dissolving 0.1 g of chitosan in 10 mL of 1% (v/v) acetic acid, followed by overnight stirring to ensure complete solubilization. The pH is then adjusted to 4.5–5 using NaOH. Separately, 0.5 g of frankincense ethanolic extract was mixed with a small volume of ethanol into the chitosan solution, followed by thorough stirring to enhance dispersion if needed. A solution of 0.05 g sodium tripolyphosphate (TPP) in 10 mL of distilled water is freshly prepared and added dropwise into the chitosan frankincense mixture under constant stirring, inducing nanoparticle formation through ionic gelation. The resulting suspension was ultrasonicated (10 min) to further reduce particle size. The formed nanoparticles are collected by centrifugation at 15,000 rpm for 15 min, washed with distilled water to remove unbound materials, and freeze-dried (or air-dried for storage).

### Physical characterization

A range of analytical techniques was employed to characterize the structural, morphological, and physicochemical properties of the chitosan–frankincense nanoparticles^33^. X-ray Diffraction (XRD analysis was performed to assess the crystalline structure of chitosan and nanoproduct. Transmission Nanoparticle morphology and size were evaluated using Electron Microscopy (TEM), an FEI TECNAI G2 F20 S-TWIN instrument (Thermo Fisher Scientific, USA). Fourier Transform Infrared Spectroscopy (FTIR) spectrum was applied for recording using a Bio-Rad FT-IR-40 spectrometer (USA). It showed characteristic absorption bands between 4000 and 500 cm^− 1^, confirming the presence of functional groups related to the components of the nanocomposite. Dynamic light scattering (DLS) was employed to evaluate the particle size distribution, polydispersity index (PDI), and zeta potential of the synthesized nanoparticles. Measurements were performed using a Malvern Zetasizer (Malvern Instruments Ltd., UK). Scanning Electron Microscopy (SEM) was used to examine the Surface morphology using a JEOL 6340 Tabletop SEM (Japan) at 15 kV, revealing the structural features of the lyophilized nanocomposite.

**The well diffusion technique**: The antimicrobial potential of Frankincense and Its Nanoparticles, both at a concentration of 20 mg/mL, was examined using the well diffusion assay^34^. This method was applied to test activity against selected microbial strains, including Gram-positive bacteria: *S. aureus* (ATCC 13565) and *S. mutans* (ATCC 25175), as well as Gram-negative strains: *E. coli* (ATCC 25922) and *S. typhi* (ATCC 14028). Antifungal efficacy was also evaluated against *C. albicans* (ATCC 10231). For bacterial analysis, nutrient agar plates were used and incubation was carried out at 37 °C for 24 h. Zones of inhibition were measured in millimeters. For fungal testing, potato dextrose agar (PDA) plates were incubated at 25 °C for 48 h before measurement of inhibition zones.

## Microplate reader assay

Minimum inhibitory concentration was assessed using a microplate-based assay, adapted with some modifications from the method described by Bechert et al., (2000)^35^. Bacterial cultures pre-adjusted to 10⁶ CFU/µL in LB broth were dispensed at 100 µL per well into a sterile 96-well microplate. Subsequently, 100 µL of each test sample, Frankincense and Its Nanoparticles, was added to the designated wells in triplicate. Plates for bacteria were incubated at 37 °C for 24 h under microaerophilic conditions, while plates for fungi were incubated at 25 °C for 48 h. Following incubation, the optical density at 620 nm was recorded using an ELISA microplate reader. The percentage of bacterial growth inhibition was quantified according to the following formula:

$${\mathrm{Inhibition}}(\% ){\text{ = }}({\mathrm{A}} - {\mathrm{A}}_{{\mathrm{1}}} ){\mathrm{/}}({\mathrm{A}}_{{\mathrm{0}}} - {\mathrm{A}}_{{\mathrm{1}}} ) \times 100.$$

Where A represents the absorbance of the treated sample, A₁ is the absorbance of the blank (medium only, without cells or treatment), and A₀ is the absorbance of the untreated control. The minimum inhibitory concentration (MIC) was defined as the lowest sample concentration that completely prevented visible bacterial growth.

### Statistical analysis

Data are expressed as mean ± standard deviation (SD) from three independent experiments, each performed in triplicate. Statistical comparisons were performed using CoStat for Windows (version 6.45). Differences were considered statistically significant when *p* < 0.05.

## Results and discussion

### Chitosan yield

The yield of chitosan obtained from shrimp shell powder was 3.4 g from 15.10 g of raw material, corresponding to a yield of 22.51%. This yield is comparable to values reported in previous studies, which typically range from 20% to 30%, depending on factors such as species, demineralization efficiency, deproteinization conditions, and degree of deacetylation. The obtained yield indicates that the extraction and purification processes were effective, suggesting that the applied method efficiently converted chitin to chitosan while minimizing material loss during processing.

### X-ray diffraction (XRD)

**Fig. 2:**
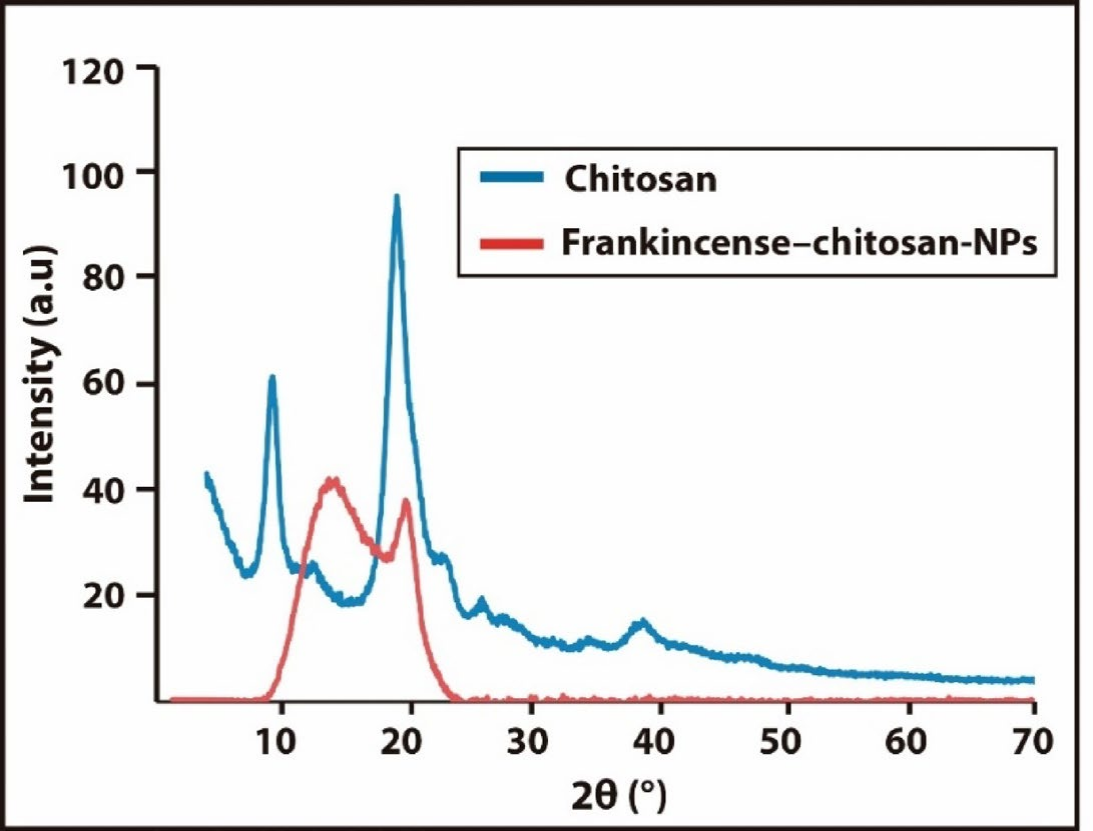
X-ray spectrum of chitosan (Blue) and frankincense–chitosan nanoparticles (Red).

Figure 2 demonstrates X-ray Diffraction (XRD) analysis which was performed to evaluate the crystalline structure of chitosan (Blue spectrum) isolated from shrimp shells. The XRD pattern of the chitosan sample displayed prominent peaks, particularly around 2θ ≈ 10° and 20°, which are characteristic of chitosan’s semi-crystalline nature. These sharp and intense peaks indicate a high degree of crystallinity, suggesting that the extracted chitosan retained well-ordered polymeric regions. The high crystal detection observed may be attributed to the specific extraction and purification conditions, which preserved the alignment of the polymer chains. This crystalline structure is important as it influences key properties such as solubility, mechanical strength, and the ability to form stable films or nanoparticles. The results confirm that the chitosan obtained from shrimp shells possesses a well-defined crystalline structure, making it a suitable candidate for further applications in drug delivery and nanotechnology^36^. The XRD pattern of frankincense–chitosan nanoparticles (Fig. 2, Red) showed broader and less intense peaks compared to pure chitosan, indicating reduced crystallinity. This suggests successful incorporation of frankincense and partial disruption of the chitosan lattice. The decrease in crystallinity may enhance solubility, stability, and controlled release of bioactive compounds. Strong interactions between chitosan and frankincense likely contribute to these structural changes. These modifications may also improve antimicrobial activity by facilitating better dispersion and cellular penetration of the nanoparticles^37,38^.

### Thermal stability (thermogravimetric analysis, TGA)

**Fig. 3:**
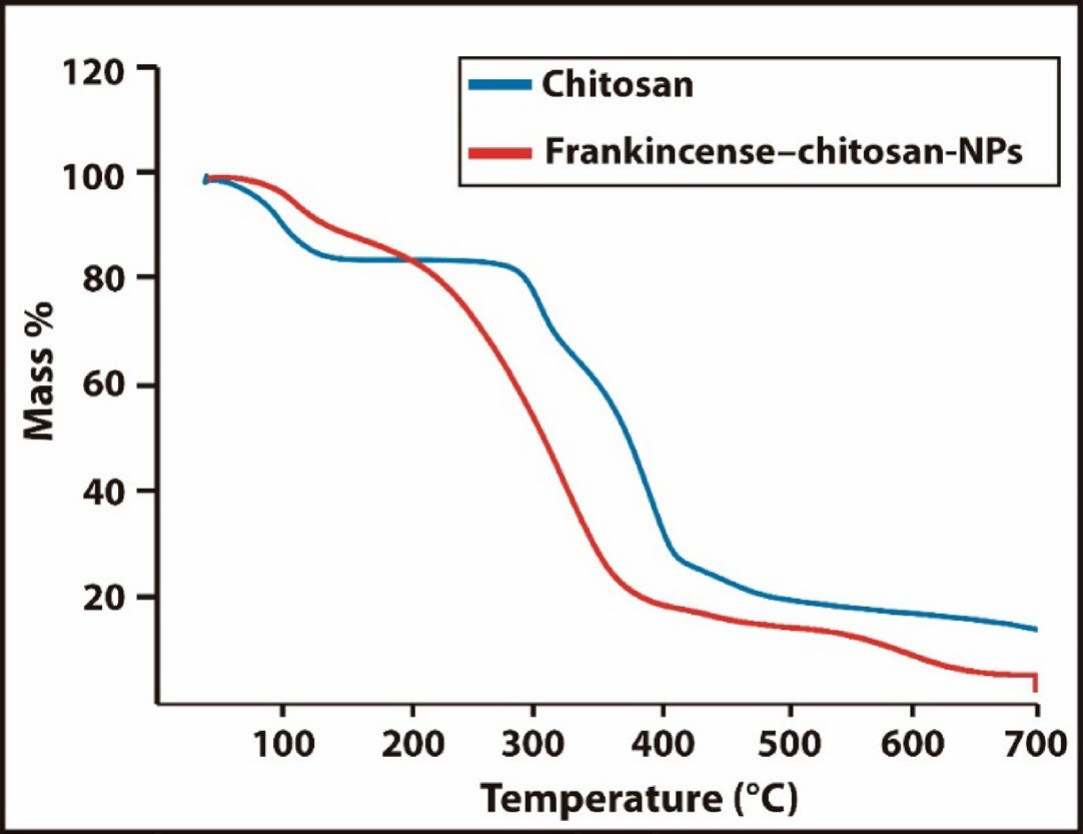
Thermogravimetric Analysis (TGA) of chitosan (Blue line) and frankincense-chitosan nanoparticles (Red line).

TGA was performed under an air atmosphere with a heating rate of 10 °C/min (Fig. 3). Both pure chitosan (Blue line) and Frankincense-chitosan nanoparticles (Frank-chitosan-NPs, Red line) exhibited an initial weight loss of approximately 10% between 51 °C and 100 °C, attributed to the loss of adsorbed moisture. For pure chitosan, the main thermal degradation occurred in two stages. The first stage, from 156 °C to 267 °C, showed a weight loss of 17.38%, likely due to polymer deacetylation and cleavage of glycosidic bonds. The second stage, from 302 °C to 460 °C, showed a larger weight loss of 78.95%, corresponding to the breakdown of the chitosan polymer backbone. This behavior is consistent with previous reports of chitosan degradation under oxidative conditions^39,40^.

In contrast, the Frank-chitosan-NPs showed slightly different thermal behavior. The first major degradation occurred from 132 °C to 382 °C with a weight loss of 71.93%, followed by a second stage from 382 °C to 445 °C with a weight loss of 83%. The broader and slightly lower temperature range of the first degradation stage suggests that the incorporation of Frankincense ethanolic extract into the chitosan matrix disrupted the polymer packing, making it less thermally stable in the initial stage^41,42^. However, the presence of bioactive compounds from the extract likely contributed to a higher residual mass and stabilization during the second degradation stage. Overall, the Frank-chitosan-NPs exhibit modified thermal behavior, reflecting the interaction between chitosan and the Frankincense extract, which affects polymer decomposition and reduces the initial structural stability^43^.

### Fourier transform infrared (FTIR) spectroscopy

**Fig. 4:**
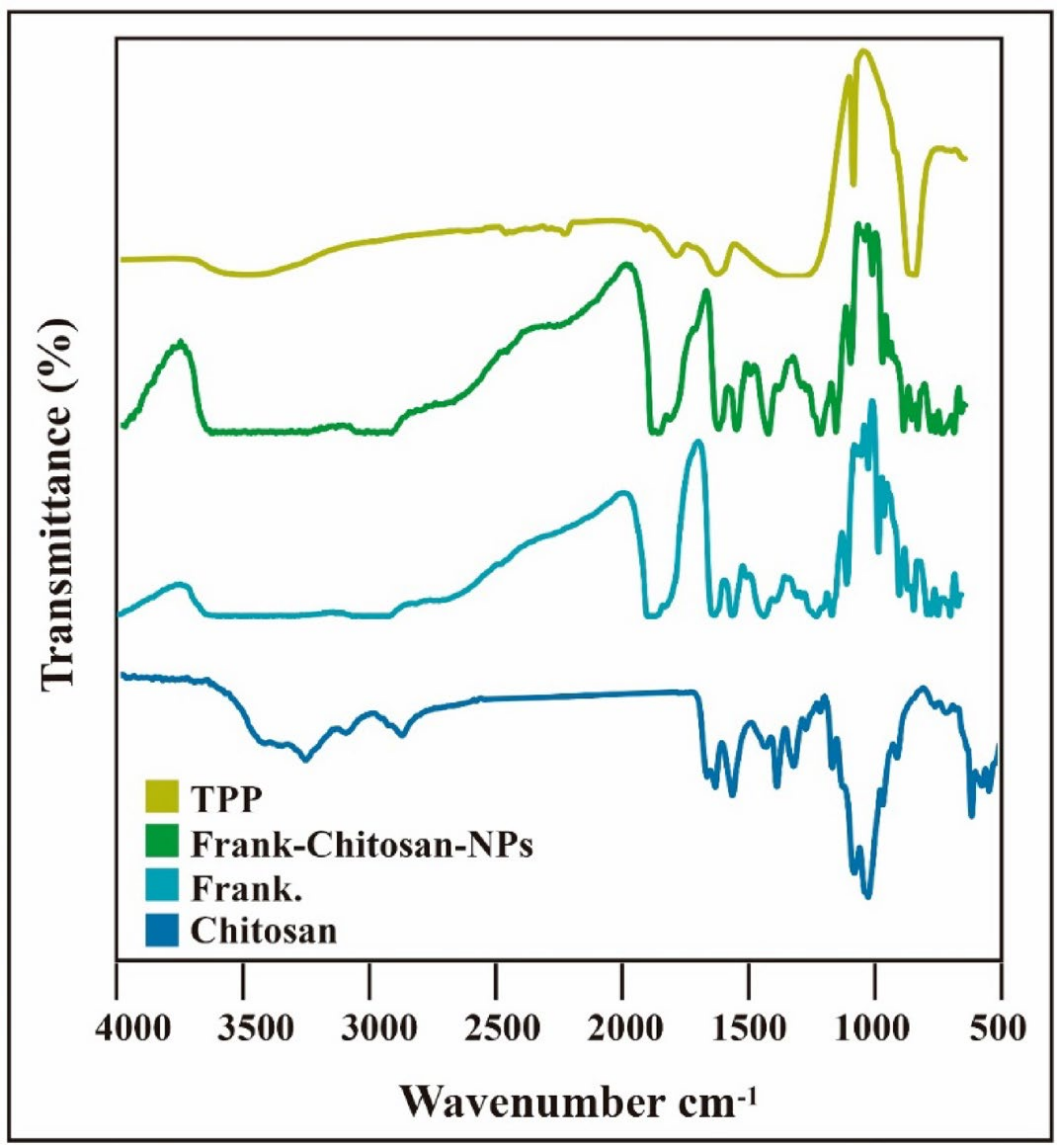
Fourier-transform infrared (FTIR) spectra of chitosan (deep blue), frankincense (Frank, teal color), sodium tripolyphosphate (TPP, olive), and Frankincense-chitosan nanoparticles (Frank-chitosan-NPs, greenish-cyan).

Figure 4 shows that the IR spectra of Chitosan (Fig. 4, Deepblue spectrum) showed a strong absorption band at 3454 cm^− 1^ due to OH and amine N–H symmetrical stretching vibrations. A peak at 2926 cm^− 1^ was due to symmetric –CH2 stretching vibration attributed to pyranose ring. The sharp peak at 1384 cm^− 1^ was assigned to –CH3 in amide group^44,45^ (amide II). The absorption bands at 1203 cm^− 1^ were assigned to the anti-symmetric stretching of C–O–C Bridge, and 1098 and 1021 cm^− 1^ were assigned to the skeletal vibrations involving the C–O stretching^46^. FTIR spectrum of the ethanolic extract of Frankincense (Fig. 4, Teal spectrum) revealed several characteristic peaks corresponding to functional groups present in its bioactive constituents. A broad and strong absorption band was observed at 3374 cm^− 1^, indicating O–H stretching vibrations typically associated with hydroxyl groups in alcohols and phenolic compounds. A distinct peak at 1736 cm^− 1^ was attributed to C=O stretching vibrations, suggesting the presence of carbonyl groups from carboxylic acids and esters, such as those found in boswellic acids^47,48^. Peaks at 1452 cm^− 1^ and 1376 cm^− 1^ correspond to bending vibrations of CH₂ and CH₃ groups, indicating aliphatic hydrocarbons. The absorption bands at 1313, 1240, 1193, 1141, 1078, and 1017 cm^− 1^ were assigned to C–O stretching vibrations, which are characteristic of alcohols, esters, and ethers^49^. Additional peaks at 950, 887, 752, and 663 cm^− 1^ were attributed to out-of-plane C–H bending of aromatic or alkene groups. The lower wavenumber region (602 to 414 cm^− 1^) exhibited multiple minor peaks, possibly related to skeletal vibrations of organic compounds and resinous structures^50^. These findings confirm the presence of diverse functional groups, supporting the complex phytochemical composition of Frankincense, particularly its content of triterpenoids and essential oil derivatives. The FTIR spectrum of sodium tripolyphosphate (Fig. 4, Olive spectrum, TPP) exhibited characteristic peaks confirming its polyphosphate structure. A strong absorption band at 1215–1250 cm^− 1^ corresponded to P=O stretching vibrations, while intense peaks at 1120–1090 cm^− 1^ and 1060–1020 cm^− 1^ were assigned to asymmetric and symmetric stretching of P–O–P linkages, respectively^51^. A distinct band near 895 cm^− 1^ indicated terminal P–O–H stretching, and a peak at around 720–715 cm^− 1^ was due to bending vibrations of P–O bonds^52^. These peaks are consistent with the polyphosphate backbone of TPP, confirming its role as a polyanionic crosslinker. The FTIR spectrum of the Frankincense-chitosan nanoemulsion (Fig. 4, GreenishCyan spectrum) exhibited characteristic absorption bands indicating the presence and interaction of both components. A broad band at 3506 cm^− 1^ corresponds to O–H and N–H stretching vibrations, suggesting hydrogen bonding between chitosan and Frankincense constituents. The peak at 2906 cm^− 1^ is due to C–H stretching of aliphatic groups. A strong absorption at 1710 cm^− 1^ is assigned to C=O stretching, likely from boswellic acids in Frankincense, while 1658 cm^− 1^ reflects amide I (C=O) of chitosan^53^. The peak at 1451 cm^− 1^ is related to CH₂ bending, and 1374 cm^− 1^ to CH₃ symmetric bending. Bands between 1239 and 1022 cm^− 1^ (1239, 1196, 1097, 1077, 1022 cm^− 1^) are due to C–O stretching and glycosidic linkages^54,55^. The peak at 950 cm^− 1^ may indicate aromatic or alkene =C–H bending. Lower-frequency bands at 662, 626, 601, 546, 525, 493, 446, and 415 cm^− 1^ are attributed to skeletal vibrations and confirm the structural complexity of the nanoparticles^56^. These spectral features confirm successful incorporation and interaction between Frankincense and chitosan.

### Transmission electron microscopy

**Fig. 5:**
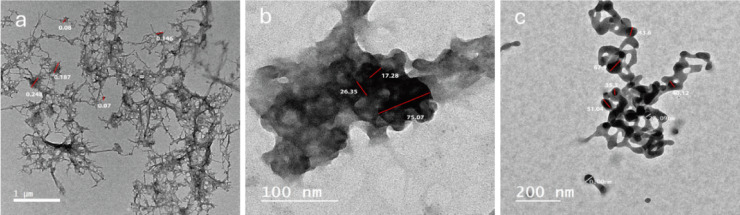
TEM images of chitosan (**a**), Frankincense extract (**b**), and Frankincense-chitosan-NPs (**c**).

The TEM images provided insight into the internal morphology of chitosan, frankincense, and their nanoparticulate formulation (Fig. 5). The TEM image of chitosan (Fig. 5a) exhibited irregular, amorphous structures with no defined boundaries, consistent with its polymeric nature. The image of frankincense (Fig. 5b) revealed particles with irregular morphology, lacking a perfectly spherical shape, and exhibiting a more aggregated distribution. In contrast, the frankincense–chitosan nanoparticles (Fig. 5c) displayed a more defined morphology with relatively uniform particle distribution and reduced aggregation, indicating successful stabilization by the chitosan matrix, with an average particle size in the range of 27–67 nm. This indicates the successful incorporation of frankincense into the chitosan matrix, resulting in a new morphology distinct from the individual components. The formation of these nanoparticles suggests improved dispersion and the potential for enhanced bioavailability and controlled release^48,57^.

### Scanning electron microscope SEM

**Fig. 6:**
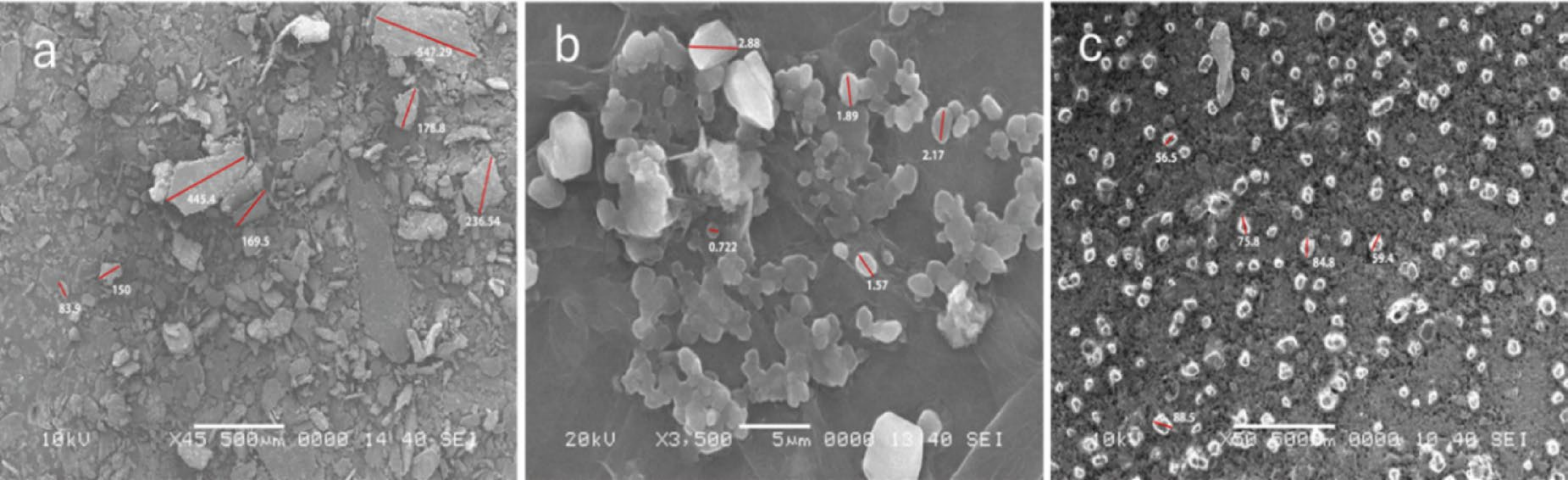
SEM images of chitosan (**a**), Frankincense extract (**b**), and Frankincense-chitosan-NPs (**c**).

Figure 6 illustrates the SEM analysis revealing distinct morphological characteristics for the individual components and the synthesized nanoparticles. Image (Fig. 6a) of pure chitosan exhibited an irregular, non-smooth, and heterogeneous surface, typical of lyophilized chitosan, which facilitates drug or phytochemical loading due to its high surface area. In contrast, the SEM image of frankincense (Fig. 6b) showed a compact, crystalline structure with uneven surfaces, reflecting its natural resinous composition. Notably, the SEM image of the frankincense-chitosan nanoparticles (Fig. 6c) demonstrated a significant morphological transformation, with the appearance of more uniform, amorphous, and reduced surface roughness. These nanoparticles were well-dispersed with sizes in the nanometer range, suggesting successful encapsulation of frankincense within the chitosan matrix. The smoother and more homogeneous surface of the composite indicates strong interaction and compatibility between frankincense and chitosan, confirming the effective formation of nanostructures suitable for enhanced bioavailability and controlled release^58^.

### Size distribution

The particle size, polydispersity index (PDI), and zeta potential of the frankincense-chitosan nanoparticles (Table 1) were determined using dynamic light scattering (DLS) via a Zetasizer. The average particle size was measured to be 493.9 ± 150 for chitosan whereas the frankincense–chitosan nanoparticles exhibited a significantly smaller size of 149.4 ± 55.8 nm, indicating the successful formation of ultra-small nanoparticles, which are advantageous for enhancing cellular uptake and improving bioavailability^59^. The PDI was found to be 0.48 for chitosan and 0.26 for frankincense–chitosan nanoparticles, suggesting a relatively broad size distribution and potential heterogeneity in the nanoparticle population. Although a lower PDI (typically < 0.3) indicates a more uniform formulation, the current value may be attributed to the natural complexity of frankincense and variations in nanoparticle formation. The zeta potential values further support the successful formation and stability of the nanoparticle systems. Chitosan alone exhibited a zeta potential of + 28.6 mV, reflecting strong cationic properties characteristic of protonated amino groups in the polymer. In comparison, the frankincense–chitosan nanoparticles showed a reduced but still positive zeta potential of + 12.0 mV, indicating a moderately stable colloidal system^60^. This decrease in surface charge suggests effective interaction and partial neutralization between chitosan and the bioactive components of frankincense during nanoparticle formation. Despite the lower charge density, the positive zeta potential remains sufficient to provide electrostatic repulsion and prevent particle aggregation^61^. Overall, these findings confirm the successful fabrication of nanoscale particles with moderate stability, making them suitable candidates for further pharmaceutical or biomedical applications, where controlled release and biocompatibility are essential considerations^62^.

**Table 1:** Particle size, polydispersity index (PDI), and zeta potential of Chitosan and Frankincense-chitosan nanoparticles.

	Chitosan	Frankincense-chitosan-NPs
Partial size (nm)	493.9 ± 150	149.4 ± 55.8
Pdi	0.48	0.26
Zeta potential (mV)	+ 28.6	+ 12.0

### Antimicrobial activity

Table 2 illustrates the antimicrobial evaluation of the extracts and nanoproducts which revealed variable effectiveness against different microbial strains. The ethanolic extract showed higher inhibition zone against *E. coli* (20 mm vs. 17.33 mm) and *S. aureus* (21 mm vs. 15 mm), likely due to the presence of volatile and hydrophobic compounds in their free form, which may act more rapidly and synergistically in the crude extract. In contrast, the nanoparticles exhibited greater activity against *Salmonella typhi* (22 mm vs. 19.33 mm), *S.mutans*. (23 mm vs. 21 mm), and *C. albicans* (23 mm vs. 20 mm), which can be attributed to the enhanced solubility, stability, and cellular penetration provided by the nano-sized droplets. These findings suggest that the choice of formulation affects antimicrobial efficacy based on the microorganism’s structural characteristics^63,64^. While nanoparticles offer improved delivery for Gram-negative bacteria and fungi with complex membranes, ethanolic extracts may retain a broader spectrum of active compounds, more effective against Gram-positive strains^65,66^.

**Table 2:** The antimicrobial evaluation of the frankincense extract and frankincense-chitosan-NPs.

	Gram negative		Gram positive		Fungi
Sample name	Inhibition zone (mm) at 20 mg/ml				
	*E.Coli*	*S.typhi*	*S.aureus*	*S.mutans*	*C.albicans*
Frankincense	20.00^b^ ± 1.00	19.33^a^ ± 1.15	21.66 ^a^ ±12.5	21.33^b^ ± 0.57	20.00^a^ ± 2.0
Frankincense-chitosan-NPs	17.33^a^ ± 0.58	22.00^a^ ± 2.00	15.00 ^b^ ±1.00	23.60 0^a^ ± 0.57	23.33^a^ ± 0.57
					
LSD	1.85	3.70	2.92	2.06	3.33

All experiments were performed in triplicate; all data are expressed as the mean ± SD. Means with different letters (in the same column) are significantly different at *p* ≤ 0.05.

### Minimum inhibitory concentrations (MIC)

**Fig. 7:**
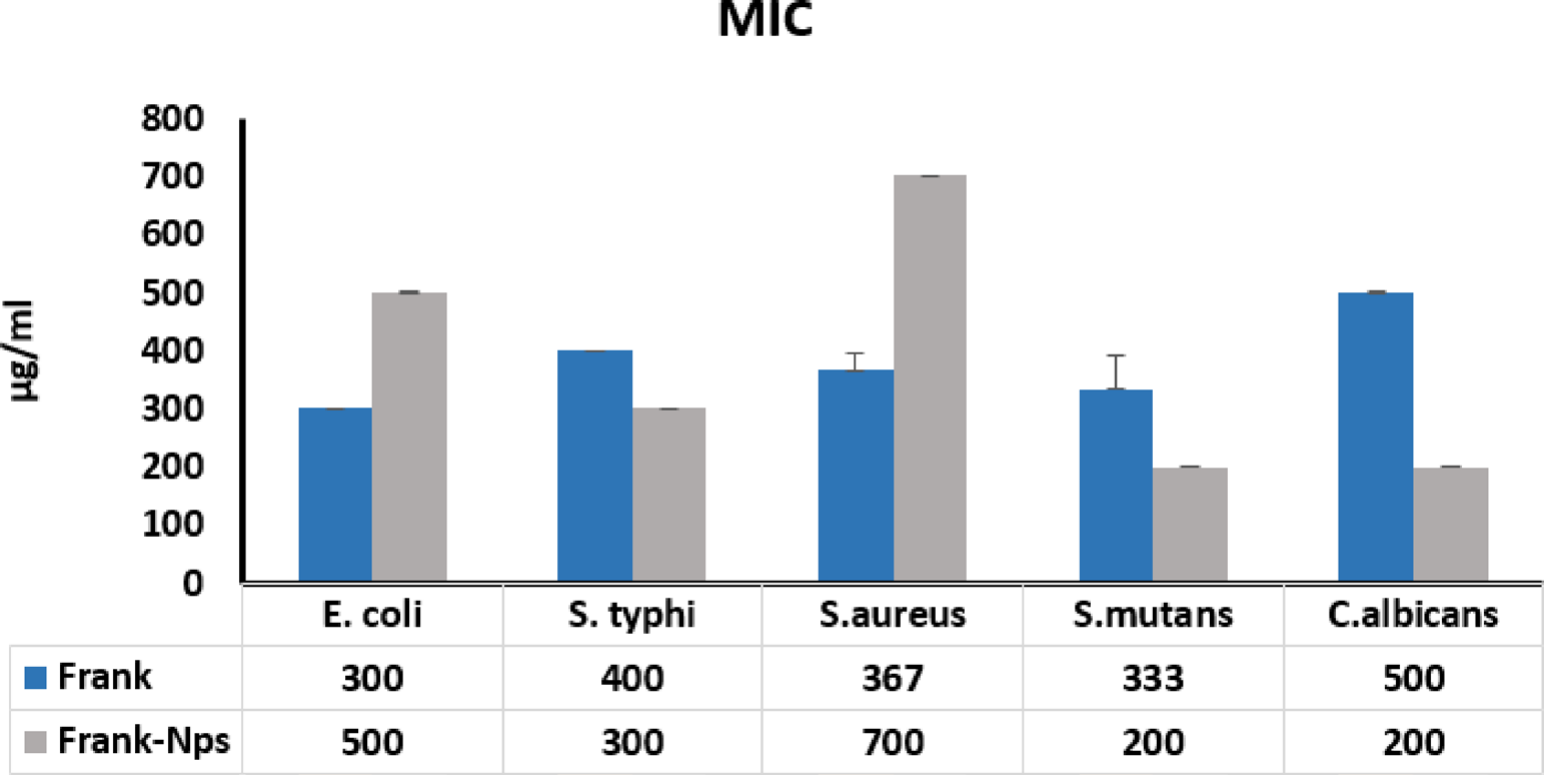
The minimum inhibitory concentrations (MICs) of the Frankincense extract (Frank) and Frankincense-chitosan-NPs (Frank-NPs).

Figure 7 demonstrates the minimum inhibitory concentrations (MICs) of frankincense ethanolic extract and its nanoparticles against various microbial strains. The data reveale distinct differences in antimicrobial effectiveness. The ethanolic extract exhibited lower MIC values against *E. coli* (300 µg/mL vs. 500 µg/mL for nanoparticles) and *S. aureus* (367 µg/mL vs. 700 µg/mL for nanoparticles), suggesting that its free-form bioactive constituents, especially volatile terpenes and lipophilic acids, exert rapid effects upon contact. This could be attributed to unencapsulated compounds directly reaching microbial targets without the need for carrier-mediated delivery, particularly benefiting Gram-positive bacteria like *S. aureus*, which possess a thick peptidoglycan layer but lack an outer membrane barrier^64,65^. In contrast, the nanoparticle formulation achieved lower MIC values against *S. Typhi* (300 µg/mL vs. 400 µg/mL for the ethanolic extract), *S. mutans* (200 µg/mL vs. 333 µg/mL), and *C. albicans* (200 µg/mL vs. 500 µg/mL). This improvement was likely due to stems from the enhanced dispersion and penetration properties. Nanoscale droplets can traverse bacterial outer membranes and fungal cell walls more efficiently than larger, unformulated compounds, facilitating sustained release and deeper cellular uptake. The increased surface area also maximizes contact between antimicrobial agents and microbial membranes, particularly beneficial for Gram-negative bacteria and fungi, where access to intracellular targets is limited by complex cell structures^67,68^.

The MICs were determined using a broth microdilution method in 96-well microplates, followed by spectrophotometric readings with a microplate reader to assess microbial growth inhibition. Overall, these findings suggest that frankincense could serve as a flexible antimicrobial agent when optimized through formulation^69^. Rather than viewing ethanolic extracts and nanoparticles as competing systems, they may be better understood as complementary tools: ethanolic extracts for rapid, direct microbial suppression, and nanoparticles for controlled delivery and enhanced interaction with resistant pathogens. This dual-strategy approach aligns with current trends in precision phytomedicine and offers a pathway toward more effective, plant-based antimicrobial therapies^48^.

## Conclusion

This study demonstrates a sustainable and efficient approach for valorizing shrimp shell waste into high-purity chitosan and employing it to fabricate frankincense-loaded nanoparticles with notable antimicrobial potential. The structural integrity of chitosan was preserved during extraction, enabling the formation of nanoscale composites with favorable morphology (average particle size 8.47 ± 1.33 nm, PDI 0.6, zeta potential + 12.9 mV) and strong functional interactions with frankincense constituents. The resulting nanoparticles exhibited enhanced antimicrobial activity, particularly against Gram-negative bacteria and fungi, with inhibition zones of 22 mm (*S. typhi*), 23 mm (*S. mutans*), and 23 mm (*C. albicans*) and MIC values ranging from 200 to 300 µg/mL. Meanwhile, the crude ethanolic extract demonstrated superior activity against Gram-positive strains, showing inhibition zones of 21 mm (*S. aureus*) and 20 mm (*E. coli*) with MICs of 367 µg/mL and 300 µg/mL, respectively. These findings highlight the complementary benefits of both systems: nanoparticles improve delivery and penetration for resistant pathogens, while crude extracts provide rapid antimicrobial effects. Overall, this work underscores the promise of natural polymer–phytochemical nanocomposites as eco-friendly antimicrobial agents and contributes to the advancement of green nanotechnology and nano-phytomedicine, laying the groundwork for future studies on stability, cytotoxicity, and in vivo performance for potential biomedical applications.

## Data Availability

All data generated or analyzed during this study are included in this published article.
