# Correction: Chitosan from shrimp shell waste as a carrier for frankincense nanoparticles with enhanced antimicrobial activity

**DOI:** 10.1038/s41598-026-40406-9

**Published:** 2026-02-19

**Authors:** Habiba A. Ahmed, Zeinab A. Salama, Abeer E. Abd El-Wahab, Ahmed M. Aboul-Enein, Amr Nassrallah

**Affiliations:** 1 Plant Biochemistry Department, National Research Centre, Dokki, Giza, 12622 Egypt; 2 Medical Biotechnology Department, Genetic Engineering and Biotechnology Research Institute, City of Scientific Research and Technological Applications, New Borg El-Arab City, Alexandria 21934 Egypt; 3 Pharmaceutical and Fermentation Industries Development Center, City of Scientific Research and Technological Applications (SRTA-City), New Borg EL-Arab, Alexandria 21934 Egypt; 4 Biochemistry Department, Faculty of Agriculture, Cairo University, Giza, 12613 Egypt; 5 Basic Applied Science Institute, Egypt-Japan University of Science and Technology (E-JUST), P.O. Box 179, New Borg El-Arab City, Alexandria 21934 Egypt

Correction to: *Scientific Reports* 10.1038/s41598-025-32342-x, published online 07 January 2026

 The original version of the Article contained an error in the Abstract, where values from Table 1 were updated prior to submission but the same values in the Abstract were not updated. As a result,

“DLS revealed an average particle size of 8.47 nm, PDI 0.6, and zeta potential + 12.9 mV”

now reads:

“DLS revealed an average particle size of 149.4 nm, PDI 0.26, and zeta potential + 12.0 mV”

The original Article has been corrected.

